# The Role of 6-Minute Walk Test Guided by Impedance Cardiography in the Rehabilitation Following Knee Arthroplasty: A Randomized Controlled Trial

**DOI:** 10.3389/fcvm.2021.736208

**Published:** 2021-11-18

**Authors:** Yangyang Lin, Xingwei Hu, Yalin Cao, Xing Wang, Yao Tong, Fengjuan Yao, Peihui Wu, Huiling Huang

**Affiliations:** ^1^Department of Rehabilitation Medicine, The Sixth Affiliated Hospital of Sun Yat-Sen University, Guangzhou, China; ^2^Department of Cardiology, Affiliated Hospital of Zunyi Medical University, Zunyi, China; ^3^Department of Cardiology, Guizhou Provincial People's Hospital, Guiyang, China; ^4^Department of Cardiology, The First Affiliated Hospital of Sun Yat-Sen University, Guangzhou, China; ^5^Department of Cardiac Ultrasound, The First Affiliated Hospital of Sun Yat-Sen University, Guangzhou, China; ^6^Department of Articular Surgery, The First Affiliated Hospital of Sun Yat-Sen University, Guangzhou, China; ^7^Key Laboratory on Assisted Circulation, Ministry of Health, Guangzhou, China

**Keywords:** rehabilitation training plan, knee arthroplasty, 6MWT, non-invasive cardiac output, ICG

## Abstract

**Objective:** To explore the effect of the 6-minute walk test (6MWT) guided by non-invasive cardiac output on the rehabilitation of patients with knee osteoarthritis following artificial total knee arthroplasty.

**Methods:** About 66 patients with knee osteoarthritis planned to undergo artificial total knee arthroplasty were included from March 2019 to October 2019, and randomly assigned to the intervention group or control group. Under the guidance of a clinical rehabilitation physician, orthopedic physician, and cardiologist, a home rehabilitation exercise program based on 6MWT and non-invasive cardiac output was formulated for patients with knee osteoarthritis. The participants of the intervention group conducted full rehabilitation training supervision and guidance through the WeChat platform to ensure their rehabilitation pieces of training were completed safely and effectively. As for the control group, patients were just given rehabilitation training manuals at the time of discharge and completed the training by themselves.

**Results:** At 6 months post-operatively, 6-minute walk distance (413.88 ± 44.61 vs. 375.00 ± 40.53 m, *P* < 0.05), active metabolic equivalent (4.13 ± 0.29 vs. 3.88 ± 0.27, *P* < 0.05), stroke volume after 6MWT (114.97 ± 12.05 vs. 98.38 ± 16.43 ml, *P* < 0.05), and cardiac output (11.92 ± 1.68 vs. 9.79 ± 1.82 l/min, *P* < 0.05) of the intervention group were significantly higher than those of the control group. The symptom evaluation scores of the intervention group were also better than those of the control group.

**Conclusions:** The multidisciplinary post-operative rehabilitation exercise training program is beneficial to the recovery of lower limb function and the improvement of exercise capacity after knee replacement, and it also helps to improve the non-invasive hemodynamic indicators related to the cardiac function of the patient.

**Clinical Trial Registration:** http://www.chictr.org.cn/index.aspx.

## Strengths and limitations of this study

▸ Ours was the first study to investigate the effect of the 6-minute walk test (6MWT) guided by the non-invasive cardiac output on the rehabilitation of patients with knee osteoarthritis undergoing artificial knee arthroplasty.
▸ We explored the feasibility and effectiveness of multidisciplinary post-operative rehabilitation guidance and follow-up based on the mobile Internet platform.
▸ A total of 36 patients were unable to return to the hospital to perform the assessment of 6MWT and the non-invasive cardiac output at 6 months due to the outbreak control of COVID-19, which may have led to bias.

## Introduction

Knee osteoarthritis (KOA) commonly occurs in the aged population, and they are also the high-risk group of cardiovascular diseases such as hypertension and coronary heart disease. The 6-minute walk test (6MWT) has been acknowledged as one of the most important methods to evaluate the cardiopulmonary function, exercise capacity, and quality of life of patients with various cardiopulmonary diseases, because of its easy implementation, safety, and low cost (1, 2). The change of 6-minute walking distance (6MWD) has been confirmed by many studies to assess the effects of drug therapy and personalized cardiopulmonary rehabilitation training (3, 4). The main technical principle of non-invasive cardiac output (CO) is thoracic bioelectrical impedance analysis. Parameters, namely, stroke volume (SV), CO, myocardial contractility index (CTI), preload rate, and peripheral vascular resistance, can be obtained through the analysis of impedance cardiography. Therefore, the hemodynamics can be monitored dynamically (5). By synchronizing non-invasive CO with 6MWT, the hemodynamic changes were detected real-time, continuously, and dynamically, to formulate a precise rehabilitation training plan.

With the development of joint surgery, rehabilitation exercise is becoming more and more important in the early stage after the operation. As an auxiliary treatment manner, post-operative rehabilitation exercise can improve the knee joint function and relieve pain timely and effectively and reduce the occurrence of complications (6). However, the effect of exercise rehabilitation after total knee arthroplasty on cardiovascular function in patients with knee osteoarthritis remains unclear. In addition, the long-term compliance of some patients is poor in traditional family rehabilitation training. With the popularity of smartphones, a variety of mobile terminal software for the popularization of health science emerged. But there are few relevant studies on whether follow-up through mobile Internet platforms can improve compliance and the effect of rehabilitation.

## Methods

### Study Design

The study was a prospective, randomized controlled trial with a follow-up of 6 months after the intervention with assessments performed to determine the role of 6MWT guided by impedance cardiography in the rehabilitation following knee arthroplasty. From March 2019 to October 2019, patients with knee osteoarthritis were screened for eligibility and invited to participate in the study before the operation in the Department of Articular Surgery of the First Affiliated Hospital of Sun Yat-sen University. Patients who met the following inclusion criteria and did not meet any of the exclusion criteria would be recruited. If the eligible patients consented to participation, they would be randomly allocated into two groups (the intervention group or the control group) in a 1:1 ratio. All participants would provide their written informed consent to participate in this study. In addition, we set up an independent outcome measurement committee to measure outcome variables and an intervener committee to implement the interventions. The study was approved by the Ethics Committees of the First Affiliated Hospital of Sun Yat-sen University, China. The study has been retrospectively registered on the Chinese Clinical Trial Registry, and the project is under approval.

Inclusion criteria (7):

1. With advanced knee osteoarthritis needs surgical treatment;
2. Between the ages of 50 and 80;
3. New York Heart Association (NYHA) grades I–III;
4. Not living alone;
5. No obvious abnormality of vital signs before operation;
6. No history of central nervous system diseases and mental disorders;
7. Normal liver and kidney function; and
8. Can communicate normally with medical staff.

Exclusion criteria:

1. Secondary osteoarthritis: such as rheumatoid arthritis, traumatic arthritis, and Charko's joint;
2. Mild joint lesion, the course <1 month, the pain visual analog scale (VAS) <4 when walking up and down stairs or squatting, or serious joint pain, VAS more than 8 when walking on a flat road;
3. Unable to walk due to severe joint disease, or suffering from other diseases that affect motor function, such as Parkinson's disease and cerebral infarction;
4. Received intra-articular injection therapy within 6 months or oral glucocorticoids within 1 month;
5. Case history of serious cardiocerebrovascular events, and/or NYHA grade worse than IV;
6. With central nervous system disease and cannot communicate normally;
7. Poorly controlled hypertension and respiratory dysfunction; and
8. Received long-term drug treatment that affected the experimental results or a history of alcohol or drug abuse.

### Central Randomization and Blinding

To avoid or reduce bias as much as possible, the randomization and allocation of intervention would be managed through a central computer network system. The random code list was generated by an independent statistician with SAS System Release 9.2 software, and which would be put into the interactive web response system (IWRS) before the experiment was applied. After eligible patients signed the informed consent form, the designated third party (a nurse) logged in to IWRS to obtain the intervention assignment. The interventions were implemented by the staff of the intervener committee. The staff of the outcome measurement committee and patients were all masked to the treatment assigned. Meanwhile, the researchers did not know which group the patients were allocated to neither did the surgeons who treated the patients during the hospital stay. As far as we knew the concealment was successful.

### Sample Size Estimation

The primary outcome variable was assessed by the 6MWT. According to the data extracted from the previous study (8), a change of 50 m in the 6MWT was considered a meaningful change in older adults. We estimated the sample size with PASS software, version 15.0, to detect a difference of 50 m between the groups. With a standard deviation of 70, a statistical power of 80%, and a two-tailed significance level of 0.05, each group had to have 30 patients. With a lost follow-up of 5%, 32 patients at least would included in each group.

### Intervention Programs

The included patients were randomly assigned into the intervention group and the control group. Unilateral artificial knee arthroplasty was performed and followed up 6 months after the operation. Under the multidisciplinary guidance of clinical rehabilitation physician, orthopedic physician, and cardiologist, a home rehabilitation exercise program that based on 6MWT and non-invasive CO was formulated for the patients with KOA (including three stages: 1–4, 4–8, and 8–24 weeks after surgery). To ensure the rehabilitation training plan was completed safely and effectively, in addition, to providing rehabilitation training manuals, the intervention group patients received full supervision and precise rehabilitation training guidance by the investigators through the Wechat platform. As for the control group, patients were just provided rehabilitation training manuals when they discharged and completed the training by themselves. The shape, color, size, and thickness of the rehabilitation training manuals were exactly the same.

### Outcome Measure

#### Primary Outcome Measure

The 6MWT was used as the primary outcome measure, it was performed before surgery and at 6 months post-operation. The changes of 6MWD, activity metabolic equivalent (METs), and exercise energy consumption were monitored. No encouragement was given to the patients during the test.

#### Secondary Outcome Measure

1. Non-invasive hemodynamic indexes: Before surgery and at 6 months post-operatively, systolic blood pressure (SBP), diastolic blood pressure (DBP), and mean arterial pressure were monitored under resting state, and heart rate, SV, CO, cardiac index (CI), CTI, left cardiac work index (LCWI), left ventricular ejection time, early diastolic filling rate (EDFR), systemic vascular resistance index (SVRI), and ejection fraction (EF) were also detected at rest and the end of the test.
2. Symptom assessment: At admission and 6 months after the operation, the pain VAS and the Western Ontario and McMaster Universities Osteoarthritis Index (WOMAC) were compared, respectively, between the two groups to evaluate the effect of rehabilitation training.

### Statistical Analyses

All statistical tests were performed using SPSS software, version 22.0. Descriptive statistics for continuous data were given as mean values with SDs (M ± SD). Normally distributed continuous data were analyzed using an independent sample *t*-test, the chi-squared test was used to analyze differences between groups for categorical data. Changes within the groups were analyzed by paired sample *t*-test. Abnormally distributed continuous data were analyzed by non-parametric Mann–Whitney U rank-sum test. Primary and secondary outcome variable analyses were conducted after the exclusion of those patients who were randomized but were found not to receive any allocated interventions or not any follow-up information (full analysis set, FAS). FAS is the main dataset of this study for the outcome evaluation. Due to the outbreak control of COVID-19, more than half of the participants were unable to return to the hospital to assess 6MWT and non-invasive cardiac output at 6 months, which would seriously affect the outcome evaluation. Thus we reported the result of 6MWT and non-invasive cardiac output based on a preprotocol analysis set. Differences were regarded as statistically significant when the two-tailed *P*-value was < 0.05.

## Results

### Study Patients

**Figure 1:**
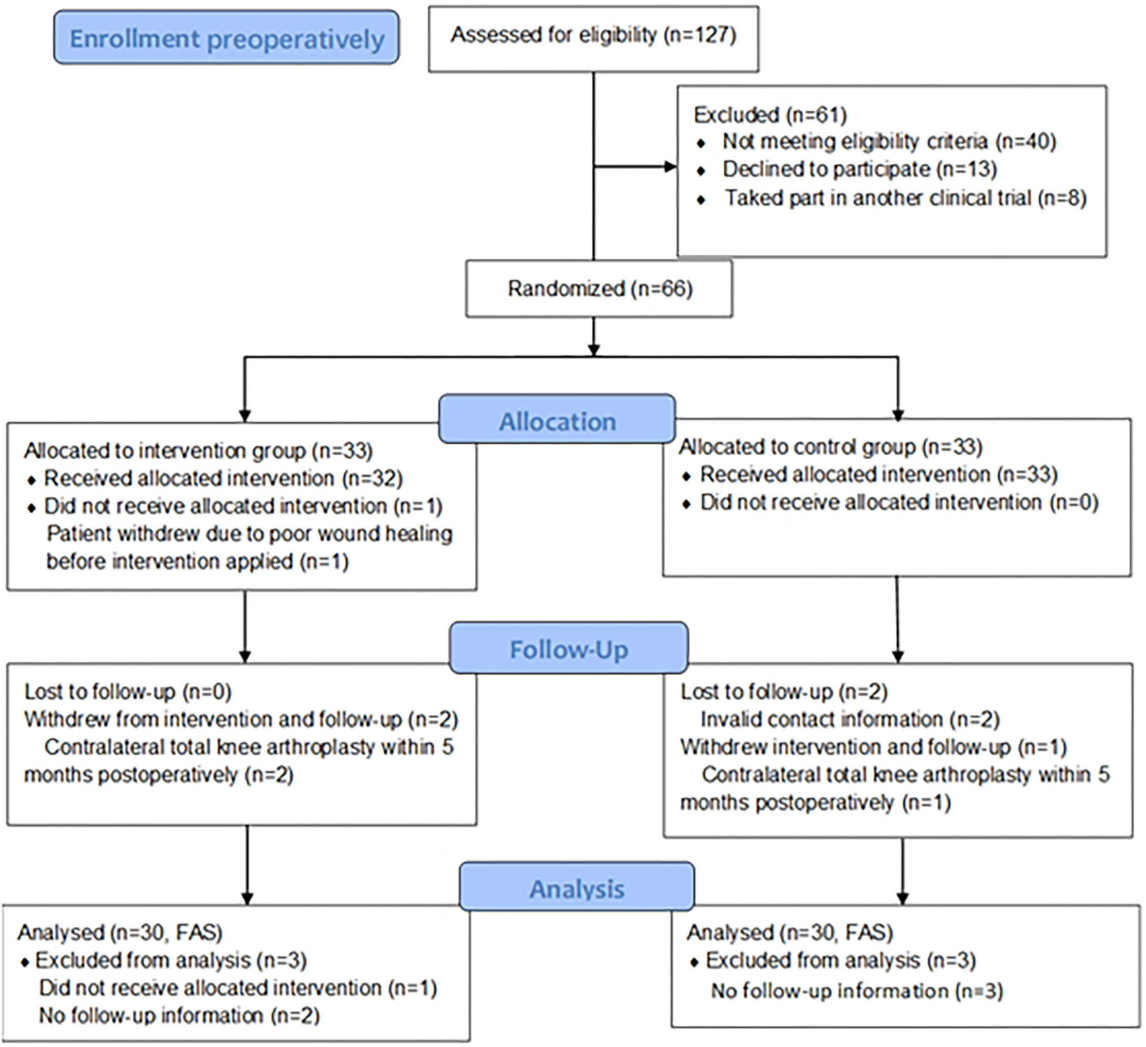
Flow diagram of the participants through the study [With reference to the CONSORT 2010 Flow Diagram, Schulz KF, et al., BMJ, 2010, 340: p. c332; (9)].

A total of 127 patients were screened for eligibility and invited to participate in our study before surgery, of whom 66 were randomized in a 1:1 ratio. Figure 1 outlines the reasons for non-recruitment. In addition to 40 patients who did not meet eligibility criteria, 13 declined to participate, eight had been taking part in another clinical trial. One patient withdrew from the study due to poor wound healing before intervention was applied. Three participants withdrew from intervention and follow-up because of contralateral total knee arthroplasty within 5 months post-operatively [*n* = 2 (6.06%) the intervention group, *n* = 1 (3.03%) the control group]. Two lost to follow-up owing to invalid contact information in the control group. Ultimately, 60 participants completed the rehabilitation training plan and follow-up. Unfortunately, due to the outbreak control of COVID-19, 18 separately in both groups were unable to return to perform the assessment of 6MWT and non-invasive cardiac output at 6 months. However, we obtained the data of VAS score and WOMAC score through a telephone follow-up survey.

### Characteristics of the Participants

General data of the included patients were collected, namely, gender, age, height, weight, body mass index (BMI), operative time, amount of blood loss, location of disease, and presence of hypertension, and there was no statistically significant difference in all above indicators (*P* > 0.05, Table 1).

**Table 1:** Characteristics of the study participants ($\bar{\text{x}}$ ± s).

	**Intervention group**	**Control group**	***t*/*X*^**2**^**	***P***
	**(*n* = 30)**	**(*n* = 30)**		
**Sex**				
Male	7	5	0.417^□^	0.519
Female	23	25		
**Age (y)**	66.16 ± 6.69	67.26 ± 7.99	−0.594^Δ^	0.555
**Height (cm)**	158.53 ± 6.03	157.32 ± 7.02	0.734^Δ^	0.466
**Weight (Kg)**	64.81 ± 9.71	61.81 ± 10.05	1.208^Δ^	0.232
**BMI (kg/m** ^**2**^ **)**	25.76 ± 3.33	24.86 ± 2.88	1.145^Δ^	0.257
**Operative time (min)**	107.28 ± 7.14	108.81 ± 7.31	−0.838^Δ^	0.405
**Blood loss (mL)**	318.75 ± 73.78	316.13 ± 58.29	0.156^Δ^	0.876
**Location of disease**				
Left	12	13	0.069^□^	0.793
Right	18	17		
**Hypertension**				
Yes	13	11	0.278^□^	0.598
No	17	19		

*Δ Indicates the value of t and □ indicates the value of chi-square*.

*BMI, body mass index*.

### Evaluation of Exercise Tolerance Between the Two Groups

At 6 months post-operatively, 6MWD, METs, and exercise energy consumption in the intervention group were significantly increased than those before surgery (*P* < 0.05), in which 6MWD and METs were better than the control group (*P* < 0.05). Moreover, the exercise energy consumption in the control group was higher after 6 months than that before operation (*P* < 0.05). Although 6MWD and METs in the control group were improved compared with pre-operation, there was no statistical difference (*P* > 0.05) (Table 2).

**Table 2:** 6MWT measures between the two groups ($\bar{\text{x}}$ ± s).

	**Intervention group**	**Control group**	***t***	***P***
	**(*n* = 12)**	**(*n* = 12)**		
**6MWD (m)**				
Pre-intervention	365.92 ± 73.41	348.92 ± 59.68	0.622	0.540
Post-intervention	413.88 ± 44.61**	375.00 ± 40.53	2.234	0.036*
**METs**				
Pre-intervention	3.81 ± 0.48	3.71 ± 0.39	0.622	0.540
Post-intervention	4.13 ± 0.29**	3.88 ± 0.27	2.243	0.035*
**Exercise energy consumption (Kcal/min)**				
Pre-intervention	4.21 ± 0.66	3.95 ± 0.75	0.901	0.378
Post-intervention	4.57 ± 0.63**	4.12 ± 0.67**	1.690	0.105

* Indicates P < 0.05 compared between groups and ** indicates within groups.

*METs, activity metabolic equivalent; 6MWD, 6-minute walking distance; 6MWT, 6-minute walking test*.

### Non-invasive Cardiac Output Indexes Between the Two Groups

(1) At rest state
  At 6 months after surgical operation, the parameters, namely, SV, CO, CI, CTI, LCWI, and EF increased and SVRI decreased compared with pre-operation in the intervention group (*P* < 0.05), and cardiac output and CI in the control group were also significantly improved (*P* < 0.05). Meanwhile, SV, CTI, and SVRI in the intervention group were better than all of them in the control group after 6 months (*P* < 0.05) (Table 3).
(2) At the end of 6MWT
  At 6 months, the indexes, namely, SV, CO, CI, LCWI, and EF in the intervention group were significantly improved compared with those before operation (*P* < 0.05), while no significant changes were found in non-invasive cardiac output in the control group (*P* > 0.05). Furthermore, SV and CI in the intervention group were higher compared with the control group after 6 months, while SVRI was lower (*P* < 0.05) (Table 4).

**Table 3:** Non-invasive cardiac output at rest ($\bar{\text{x}}$ ± s).

	**Intervention group**	**Control group**	***t*/*Z***	***P***
	**(*n* = 12)**	**(*n* = 12)**		
**HR (min** ^**−1**^ **)**				
Pre-intervention	73.17 ± 8.24	85.50 ± 23.20	−1.389^□^	0.097
Post-intervention	78.50 ± 11.53	80.50 ± 10.98	−0.435Δ	0.668
**SV (ml)**				
Pre-intervention	71.85 ± 14.21	67.53 ± 12.54	0.770Δ	0.450
Post-intervention	82.13 ± 10.06**	68.85 ± 10.91	3.100Δ	0.005*
**CO (L/min)**				
Pre-intervention	5.14 ± 1.39	5.08 ± 0.89	0.140Δ	0.890
Post-intervention	6.51 ± 1.56**	5.48 ± 0.78**	2.057Δ	0.052
**CI [(L/(min** **^−1^·** **m** ^**−2**^ **)]**				
Pre-intervention	3.12 ± 0.85	3.15 ± 0.67	−0.107Δ	0.916
Post-intervention	3.89 ± 0.90**	3.38 ± 0.65**	−1.389^□^	0.165
**CTI (dPmx)**				
Pre-intervention	152.68 ± 55.25	163.38 ± 57.54	−0.465Δ	0.647
Post-intervention	193.55 ± 66.10**	138.52 ± 51.17	−2.194^□^	0.028*
**LCWI (kg** **×** **m** **×** **m**^**−2**^**)**				
Pre-intervention	4.20 ± 1.79	4.79 ± 1.36	−1.388Δ	0.165
Post-intervention	5.47 ± 1.63**	4.68 ± 1.22	1.164Δ	0.257
**LVET (ms)**				
Pre-intervention	400.91 ± 58.78	376.66 ± 57.78	−0.981^□^	0.326
Post-intervention	373.38 ± 63.90	361.22 ± 78.37	0.417Δ	0.681
**EDFR (%)**				
Pre-intervention	56.00 ± 5.80	54.77 ± 10.89	−1.790^□^	0.073
Post-intervention	62.97 ± 13.07	62.69 ± 12.70	0.052Δ	0.959
**SVRI (D.S** **×** **cm**^**−5**^ **×** **m**^**−2**^**)**				
Pre-intervention	2456.83 ± 435.38	2484.42 ± 334.83	−0.174Δ	0.863
Post-intervention	2041.58 ± 429.25**	2372.33 ± 337.18	−2.099Δ	0.048*
**EF (%)**				
Pre-intervention	55.07 ± 10.13	56.71 ± 9.18	−0.493^□^	0.488
Post-intervention	59.06 ± 10.58**	53.67 ± 10.80	−1.126^□^	0.260

Δ Indicates the value of t, □ indicates Z value of Mann–Whitney U rank-sum test; * indicates P < 0.05 between-group comparison and ** indicates within-groups.

**Table 4:** Non-invasive cardiac output at the end of 6MWT ($\bar{\text{x}}$ ± s).

	**Intervention group**	**Control group**	***t*/*Z***	***P***
	**(*n* = 12)**	**(*n* = 12)**		
**HR (min** ^**−1**^ **)**				
Pre-intervention	111.08 ± 25.74	100.50 ± 21.475	1.094Δ	0.286
Post-intervention	104.08 ± 13.56	101.00 ± 11.74	0.595Δ	0.558
**SV (ml)**				
Pre-intervention	92.30 ± 19.67	90.98 ± 15.45	0.182Δ	0.857
Post-intervention	114.97 ± 12.05**	98.38 ± 16.43	2.820Δ	0.010*
**CO (L/min)**				
Pre-intervention	9.99 ± 3.25	9.68 ± 1.63	0.301Δ	0.766
Post-intervention	11.92 ± 1.68**	9.79 ± 1.82	2.978Δ	0.007
**CI [(L/(min** **^−1^·** **m** ^**−2**^ **)]**				
Pre-intervention	5.98 ± 1.93	6.06 ± 1.18	−0.115Δ	0.910
Post-intervention	7.15 ± 1.04**	6.11 ± 1.17	2.299Δ	0.031*
**CTI (dPmx)**				
Pre-intervention	269.33 ± 124.11	294.18 ± 145.52	−0.450Δ	0.657
Post-intervention	353.63 ± 124.43	283.66 ± 105.78	1.484Δ	0.152
**LCWI (kg** **×** **m** **×** **m**^**−2**^**)**				
Pre-intervention	8.03 ± 2.95	8.18 ± 2.55	−0.133Δ	0.895
Post-intervention	9.83 ± 2.08**	8.44 ± 2.39	1.515Δ	0.144
**LVET (ms)**				
Pre-intervention	274.28 ± 116.23	294.18 ± 63.43	0.594Δ	0.559
Post-intervention	235.63 ± 73.19	227.83 ± 81.16	0.247Δ	0.807
**EDFR (%)**				
Pre-intervention	75.89 ± 14.71	65.33 ± 21.50	1.404Δ	0.174
Post-intervention	67.93 ± 19.24	64.97 ± 22.18	−0.404^□^	0.686
**SVRI (D.S** **×** **cm**^**−5**^ **×** **m**^**−2**^**)**				
Pre-intervention	1572.50 ± 685.65	1290.25 ± 205.57	1.366Δ	0.186
Post-intervention	1125.42 ± 245.45	1337.42 ± 228.479	−2.190Δ	0.039*
**Ejection Fraction (EF) (%)**				
Pre-intervention	62.36 ± 11.77	64.22 ± 11.75	−0.387Δ	0.702
Post-intervention	69.02 ± 9.26**	63.68 ± 12.94	1.163Δ	0.257

Δ Indicates the value of t, □ indicates Z value of Mann–Whitney U rank-sum test; * indicates P < 0.05 compared between groups and ** indicates within groups.

*CO, cardiac output; CI, cardiac index; CTI, contractility index; EDFR, early diastolic filling rate; EF,; HR, heart rate; LCWI, left cardiac work index; LVET, left ventricular ejection time; SV, stroke volume; SVRI, systemic vascular resistance index*.

### Symptom Assessment

**Figure 2:**
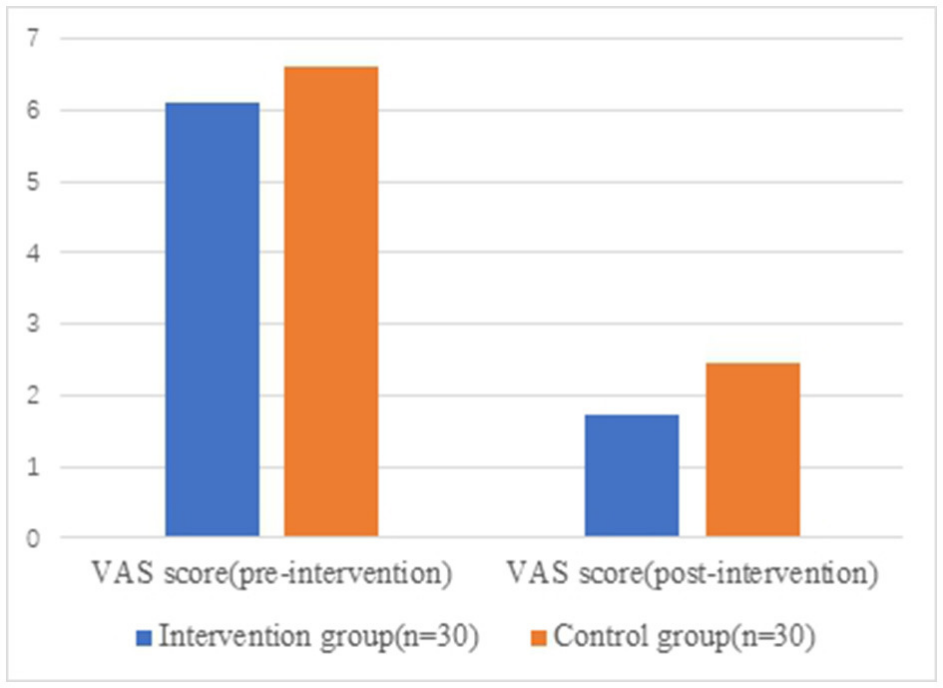
VAS score between groups. VAS, visual analog scale.

**Figure 3:**
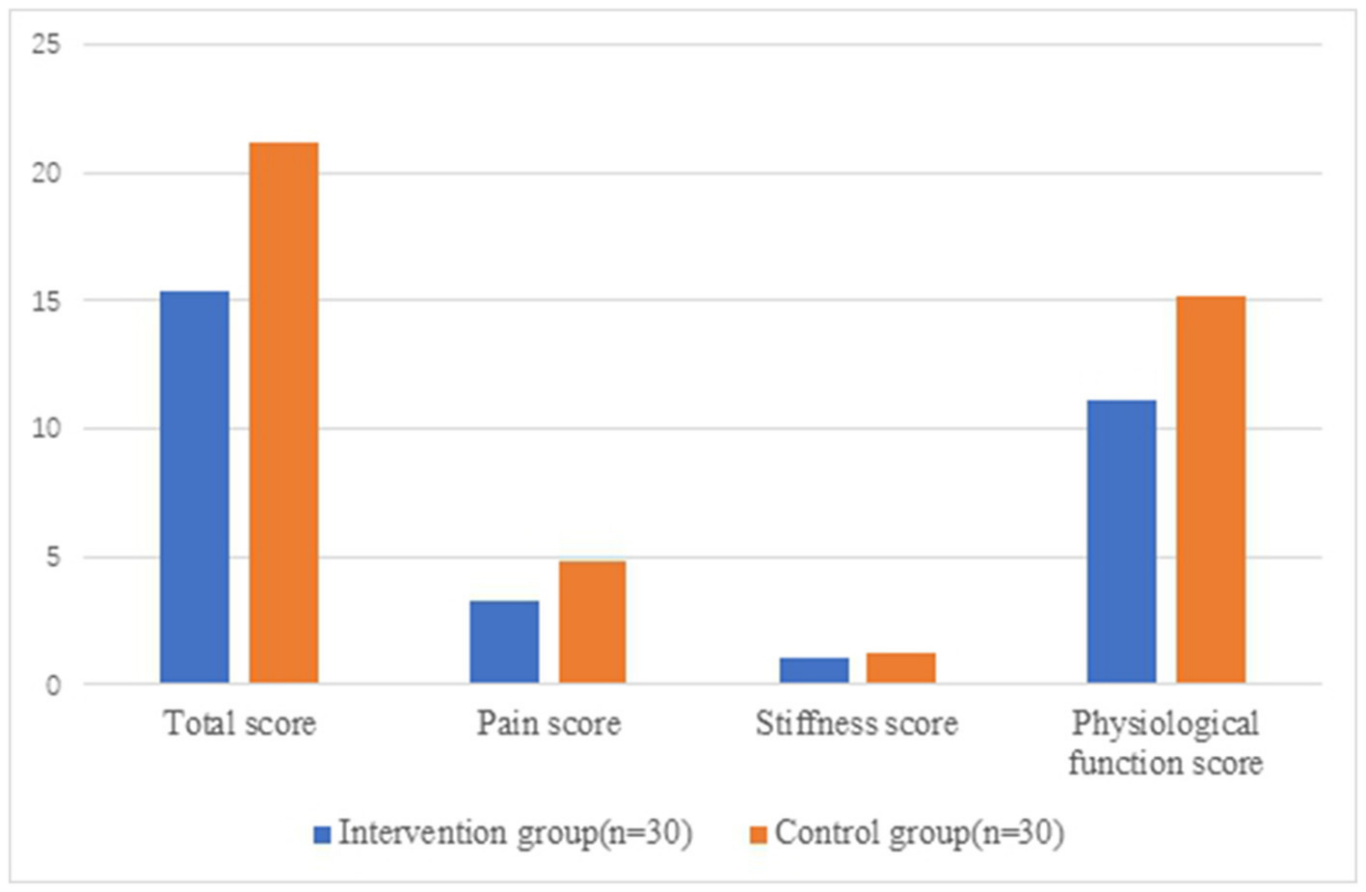
WOMAC score measure between groups post-intervention. WOMAC, Western Ontario and McMaster Universities Osteoarthritis Index.

There were no significant differences in VAS score, total score, and each score of WOMAC between the two groups before surgery (*P* > 0.05). After 6 months post-operatively, the VAS score and WOMAC score within both groups were better than those before surgical operation (*P* < 0.05), and all of VAS score, WOMAC score, namely, total score, pain score, and physiological function score in the intervention group significantly preceded the control group (*P* < 0.05) (Tables 5, 6 and Figures 2, 3).

**Table 5:** VAS score between groups ($\bar{\text{x}}$ ± s).

	**Intervention group**	**Control group**	***Z***	***P***
	**(*n* = 30)**	**(*n* = 30)**		
**VAS score**				
Pre-intervention	6.10 ± 1.03	6.60 ± 1.25	−1.922	0.055
Post-intervention	1.73 ± 1.05	2.47 ± 1.33	−2.25	0.024*
*Z*	−4.825	−4.719	–	–
*P*	0.000**	0.000**	–	–

* Indicates P < 0.05 compared between groups and ** indicates within groups.

*VAS, visual analog scale*.

**Table 6:** WOMAC score measure between groups ($\bar{\text{x}}$ ± s).

	**Intervention group**	**Control group**	***t*/*Z***	***P***
	**(*n* = 30)**	**(*n* = 30)**		
**WOMAC (Total score)**				
Pre-intervention	56.90 ± 10.92	59.53 ± 12.42	−0.872Δ	0.387
Post-intervention	15.43 ± 8.42**	21.17 ± 8.88**	−2.566Δ	0.013*
**Pain score**				
Pre-intervention	12.90 ± 2.62	13.87 ± 2.96	−1.341Δ	0.185
Post-intervention	3.27 ± 1.95**	4.80 ± 2.78**	−2.472Δ	0.016*
**Stiffness score**				
Pre-intervention	1.33 ± 0.80	1.47 ± 0.90	−0.440^□^	0.660
Post-intervention	1.07 ± 0.91	1.20 ± 0.89	−0.529^□^	0.597
**Physiological function score**				
Pre-intervention	42.67 ± 10.52	44.20 ± 10.31	−0.570Δ	0.571
Post-intervention	11.10 ± 7.24**	15.17 ± 7.21**	−2.180^□^	0.033*

Δ Indicates the value of t, □ indicates Z value of Mann–Whitney U rank-sum test; *indicates P < 0.05 compared between groups and **indicates within groups.

*WOMAC, Western Ontario and McMaster Universities Osteoarthritis Index*.

## Discussion

For patients undergoing knee arthroplasty, the purpose of post-operative rehabilitation exercise is mainly to adapt to the prosthesis, prevent joint stiffness, increase the range of motion, and promote the recovery of function. In addition, rehabilitation exercise also has a certain positive effect on cardiopulmonary function and systemic inflammatory response. In our study, under the multidisciplinary guidance of clinical rehabilitation physician, orthopedic physician, and cardiologist, a home rehabilitation exercise program that based on 6MWT synchronized with non-invasive cardiac output was formulated for the patients with KOA, and the influence of post-operative rehabilitation exercise on cardiac and lower limb function of patients with KOA, and how to formulate an appropriate post-operative treatment, follow-up and rehabilitation training plan to make operation to achieve the best therapeutic effect were explored.

### Assessment of Activity Tolerance

In addition to reflecting the cardiac function of patients under submaximal exercise, 6MWT is also used to evaluate the activity tolerance and lower limb function. A study suggests that low muscle strength and joint pain of the lower extremity may lead to slow walking speed and short 6MWD during 6MWT (10). Other studies have demonstrated that 6MWT has good reliability and validity in the assessment of motor function and activity tolerance in patients with lower limb arthritis and joint replacement (11, 12).

In this study, we found that 6MWD, METs, and exercise energy consumption of patients in the intervention group significantly increased compared with pre-intervention after 6 months of rehabilitation exercise (*P* < 0.05), and 6MWD and METs were better than the control group (*P* < 0.05).

Patients with knee osteoarthritis commonly tend to develop bad habits such as being sedentary and bedridden because of lower limb pain, and their daily activity was significantly lower than that of normal people (13). And patients who underwent artificial knee arthroplasty will be able to walk longer distances, have better exercise capacity, and can tolerate more activity through rehabilitation training. During the 6 months of post-operative rehabilitation training, the exercise tolerance and motor function were significantly improved, enabling the patients to do more and longer exercise, and obtain a virtuous cycle. In addition, the improvement of walking distances and METs in the intervention group were better than those in the control group, which indicates that rehabilitation exercise under professional guidance and supervision can help patients develop good training habits and maintain a certain amount of daily exercise, and contribute to complete the training plan on time, and improve their activity tolerance.

### Evaluation of Non-invasive Cardiac Output

Patients with lower extremity joint disease may fail to reach the submaximal exercise state due to low limb pain during 6MWT, which will affect the evaluation of cardiac function. The post-operative 6MWD of the patients in the intervention group increased significantly, should this be attributed to the improvement of cardiac function or the recovery of lower limb function? Obviously, the traditional 6MWT has some limitations.

Non-invasive cardiac output detection technology, also known as non-invasive hemodynamic monitoring technology, which main technical principle is thoracic electrical bioimpedance. As the heart relaxes and contracts, the intravascular blood flow changes, and the impedance when the current passes the chest also change accordingly. Non-invasive hemodynamic monitoring by impedance cardiography is based on the principle of the impedance of the thorax, parameters, namely, SV, CO, CTI, EDFR, SVR, etc. could be obtained by processing impedance cardiogram, and these parameters could be detected dynamically and continuously. By synchronizing impedance cardiography, non-invasive hemodynamic monitoring with 6MWT, the changes in cardiac function can be more accurately reflected during 6MWT.

In our research, it was found that in addition to the increase of CO, CI, CTI, EF, and LCWI, SVRI was also significantly decreased at 6 months after operation in the intervention group. We held the opinion that, with the improvement of lower limb function after the operation, patients gradually increased the amount of daily activity and carried out rehabilitation exercises as required, which was conducive to the improvement of physical fitness and cardiac function. Besides the improvement of the strength and coordination of lower limb muscles by the training exercise itself, deep breathing, abdominal and chest breathing training would contribute to enhancing the strength of respiratory muscles, expanding the range of thoracic movement, and increasing the distance of the diaphragm downward in the process of post-operative rehabilitation training, which could effectively expand the airway, reduce respiratory resistance. And then the circulating blood volume also increased when the ventilation-perfusion ratio remained constant, further improving cardiac function, consequently. Furthermore, rehabilitation exercise could promote venous blood reflux caused by muscle contraction, which increased circulating blood volume. It was basically consistent with the results of Wang et al. (14).

At the end of the trial, CI and CO in the intervention group were higher than those in the control group, while SVRI was lower. And EF and LCWI were also increased compared to pre-operation (*P* < 0.05). It suggested that multidisciplinary exercise prescription based on hemodynamics and post-operative rehabilitation guidance on the strength of mobile Internet platforms were helpful to the recovery of cardiac function. Some scholars have proposed that patients with hip or knee arthritis could not significantly improve their daily activities after joint replacement (15). In fact, the effect of traditional family rehabilitation training is limited by the own educational level, compliance, and understanding of rehabilitation training. And the effect of a post-operative rehabilitation training plan with professional supervision and guidance is better than the traditional family rehabilitation training.

In the control group, there was no significant difference in the changes of non-invasive cardiac output between pre-operation and post-operation, which may be associated with the lack of guidance in traditional family rehabilitation training. Although the daily activities of patients have improved after surgical operation, insufficient training time and intensity, lack of aerobic exercise training, and irregular functional training on the improvement of cardiac function are limited.

### Improvement of Symptoms

VAS and WOMAC scores are commonly used to evaluate the subjective feelings of patients with osteoarthritis. VAS score is mainly used for the overall evaluation of pain symptoms of patients, and the WOMAC scale is divided into three dimensions, namely, pain, joint stiffness, and dysfunction (16, 17). Pain and limitation of physiological function are the main reasons for most patients with osteoarthritis to seek medical treatment. After joint functional exercise and moderate aerobic exercise, patients with osteoarthritis were evaluated by WOMAC, KOOS, and other rating scales again, and their scores were found to be improved (18). Domestic scholars have verified the effectiveness of using the WOMAC scale to evaluate the pathogenetic condition of Chinese patients with knee arthritis (19).

In this study, the subjective pain assessment of patients in both groups had been significantly improved after rehabilitation training (*P* < 0.05), and the VAS score of the intervention group was better than that of the control group, and the difference was statistically significant (*P* < 0.05). Meanwhile, the pain score and function score of WOMAC in the intervention group were better than those in the control group (*P* < 0.05), which was basically consistent with the results of previous studies.

Moreover, combined with the indexes of non-invasive cardiac output, this study showed that patients with knee osteoarthritis were treated with surgery and rehabilitation training, even though some patients had poor compliance and did not complete the rehabilitation training as planned, it is also possible to improve their exercise tolerance and cardiac function by increasing the amount of daily activity. And the score of rating scales given by the patients in the intervention group was better, which indicated that rehabilitation training under professional supervision could make patients obtain a better quality of life and be more satisfied with the surgical effect.

## Conclusions

To formulate a precise rehabilitation training plan with 6MWT guided by non-invasive cardiac output is beneficial to the recovery of lower limb function and the increase of exercise tolerance after knee arthroplasty, and the improvement of cardiac function and quality of life. The accurate rehabilitation exercise prescription based on the guidance of clinical rehabilitation physician, orthopedic physician, and cardiologist is worth applying and popularizing for patients underwent knee arthroplasty.

## Data Availability Statement

The raw data supporting the conclusions of this article will be made available by the authors, without undue reservation.

## Ethics Statement

The studies involving human participants were reviewed and approved by the Ethics Committees of the First Affiliated Hospital of Sun Yat-Sen University, China, approved the study (No. [2019]259). The patients/participants provided their written informed consent to participate in this study.

## Author Contributions

HH and PW conceived of the study. YL and XH performed the statistical analysis and drafted the manuscript. HH, PW, YL, XH, and YC developed the study protocol. XW, YT, and FY led the implementation of the study and collected data. YC, XW, and YT performed follow-up for all patients. All authors revised it critically for important intellectual content and approved the final manuscript.

## Funding

This study was supported by the Open Project of State Key Laboratory of Organ Failure Research (Grant No. G820NF1024).

## Conflict of Interest

The authors declare that the research was conducted in the absence of any commercial or financial relationships that could be construed as a potential conflict of interest.

## Publisher's Note

All claims expressed in this article are solely those of the authors and do not necessarily represent those of their affiliated organizations, or those of the publisher, the editors and the reviewers. Any product that may be evaluated in this article, or claim that may be made by its manufacturer, is not guaranteed or endorsed by the publisher.
